# A new experimental setup for combined fast differential scanning calorimetry and X-ray photon correlation spectroscopy

**DOI:** 10.1107/S1600577524002510

**Published:** 2024-04-24

**Authors:** Alessandro Martinelli, Jacopo Baglioni, Peihao Sun, Francesco Dallari, Eloi Pineda, Yajuan Duan, Tobias Spitzbart-Silberer, Fabian Westermeier, Michael Sprung, Giulio Monaco

**Affiliations:** a University of Padova, Department of Physics and Astronomy ‘Galileo Galilei’, Via F. Marzolo 8, 35131 Padova, Italy; b Department of Physics, Institute of Energy Technologies, Universitat Politècnica de Catalunya – BarcelonaTech, 08019 Barcelona, Spain; c Deutsches Elektronen-Synchrotron DESY, Notkestraße 85, 22607 Hamburg, Germany; Advanced Photon Source, USA

**Keywords:** X-ray photon correlation spectroscopy, fast differential scanning calorimetry

## Abstract

Synchrotron-radiation-based techniques are a powerful tool for the investigation of materials. In particular, the availability of highly brilliant sources has opened the possibility to develop techniques sensitive to dynamics at the atomic scale such as X-ray photon correlation spectroscopy (XPCS). XPCS is particularly relevant in the study of glasses, which have been often investigated at the macroscopic scale by, for example, differential scanning calorimetry. Here, we show how to adapt a Flash calorimeter to combine XPCS and calorimetric scans. This setup paves the way to novel experiments requiring dynamical and thermodynamic information, ranging from the study of the crystallization kinetics to the study of the glass transition in systems that can be vitrified thanks to the high cooling rates reachable with an ultrafast calorimeter.

## Abstract 2

A commercial fast scanning calorimeter is adapted to a synchrotron radiation beamline to perform experiments of both X-ray photon correlation spectroscopy and calorimetry. The capabilities of the setup are discussed in light of results obtained on both oxide and metallic glasses.

## 1. Introduction

Synchrotron-radiation-based techniques are a state-of-the-art tool for the investigation of matter at the microscopic scale. In the last 30 years, they have led to outstanding advances in the fields of materials science, chemistry, physics and biology, and are nowadays routinely utilized by scientists given their versatility and availability (Willmott, 2019 ▸). The use of X-rays has well known advantages. First of all, their wavelengths (∼Å) match the typical interatomic distances in condensed matter systems, thereby enabling studies of materials with atomic resolution. In addition, the strong dependence of X-ray absorption on the atomic number has led to the development of powerful imaging techniques.

Although X-rays are routinely employed in laboratory-based setups, the exceptional brilliance of synchrotron radiation sources has enabled many new applications, including X-ray nano-diffraction, nuclear scattering, inelastic X-ray scattering, time-resolved diffraction and X-ray photon correlation spectroscopy (XPCS). XPCS, specifically, is a technique that has particularly benefited from recent developments of X-ray sources and grants direct access to the real-time atomic dynamics in disordered systems (Sandy *et al.*, 2018 ▸; Perakis & Gutt, 2020 ▸; Madsen *et al.*, 2020 ▸). In this scattering method, a partially coherent X-ray beam is diffracted by density fluctuations in the sample under study and the measured intensity of the scattered X-rays shows a peculiar interference pattern, known as a speckle pattern, whose time evolution embeds information on the dynamics of the sample under study. Despite the great attractiveness of XPCS results, many physical processes of current interest in materials science require, for a deeper understanding, the simultaneous collection of data from complementary techniques. In glass science, calorimetric scans are particularly informative and therefore play a central role (Zheng *et al.*, 2019 ▸). In fact, the glass transition is a kinetic process where a liquid cooled below its melting temperature reaches eventually an amorphous ‘frozen’ state. When heating the glass, clear signatures are detectable with calorimetry: the difference in specific heat capacity between the liquid and the glass (essentially due to the ‘freezing’ of the translational degrees of freedom) is reflected in a jump in the heat flux recorded in the calorimetric measurement at the glass transition, and depends on the thermal history of the glass.

Recently, thermodynamic properties measured by chip-based nano-calorimeters have been investigated in combination with X-ray diffraction employing both micro- and nano-focused beams (Xiao *et al.*, 2013 ▸; Rosenthal *et al.*, 2014 ▸). Such setups are very appealing for applications where the sample quantity is limited (for example, pharmaceutical or biological systems that can be produced only in reduced amounts). In glass science, this combination of an X-ray based technique with a nano-calorimeter provides attractive advantages since the properties of the sample depend on the protocol used for its preparation and nano-calorimeters permit controlled and reproducible temperature cycles. Furthermore, nano-calorimeters can achieve rates as high as 10^6^ K s^−1^ (Yi & LaVan, 2019 ▸), and this in fact gives the possibility to prepare (and study) many materials prone to crystallization, such as metallic glasses or polymers (Schawe & Pogatscher, 2016 ▸). In this paper, we show how to adapt a commercial chip-based calorimeter (Flash DSC 2+ from Mettler Toledo) and integrate it in a coherent scattering beamline. This setup can be utilized efficiently to perform XPCS experiments in combination with calorimetric scans, and we present here the possibilities opened by this development.

## 2. X-ray photon correlation spectroscopy

In XPCS, an X-ray beam impinges on the sample and the scattered X-ray intensity *I*(**q**, *t*_1_) is recorded as a function of time *t*_1_ and momentum transfer *q* = 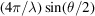 , with λ being the X-ray wavelength and θ the scattering angle. Then, the correlation function is calculated between pairs of intensities separated by a delay *t*,

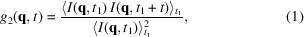

where the angular brackets indicate the average over the initial time *t*_1_. Here it is assumed that the dynamics are independent or weakly dependent on *t*_1_ during the course of the measurement. In the following, under the assumption that the supercooled liquids and the glasses to be discussed hereafter have an isotropic dynamical response, we will drop the vector notation for *q*. While this assumption is likely to be correct, we have no simple way to test it here as we only probe *q*-values along one direction as selected by the range of accessible scattering angles, as detailed hereafter.

In the Gaussian approximation for the scattered field (Berne & Pecora, 1976 ▸), *g*_2_(*q*, *t*) is directly related to the square modulus of the intermediate scattering function, *F*(*q*, *t*),

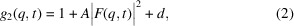

where the intermediate scattering function *F*(*q*, *t*) is the auto-correlation function of the *q*-component of the microscopic density, *d* is a baseline arising from the non-uniform illumination of the detector and *A* is a coefficient, known as *contrast*, which depends on the coherence properties of the X-ray beam and on the scattering geometry (Madsen *et al.*, 2020 ▸). In glasses and supercooled liquids, the intermediate scattering function is usually characterized by different relaxation processes (Monaco *et al.*, 1998 ▸, 1999 ▸), among which the slowest one is called the structural relaxation. This relaxation is usually modeled using the empirical Kohlrausch–Williams–Watts (KWW) function (Williams & Watts, 1970 ▸), 

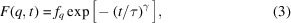

with *f*_*q*_ denoting the strength of the process, τ the relaxation time and γ the shape parameter. These parameters depend, in general, on the momentum transfer *q*. For notational simplicity, in the following we will use an ‘effective’ contrast *c* = 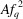 . The use of hard X-rays allows access to high *q* values which correspond to interatomic distances. By changing the scattering angle, XPCS offers then the possibility to cover length scales from micrometres down to sub-nanometres. For what concerns the accessible time range, XPCS measurements carried out with the detector used here (EigerX4M) can reach a minimum time of the order of few milliseconds. The maximum time scale is dictated instead by the stability of the experimental setup or by effects of radiation damage in the sample, and can exceed thousands of seconds (Madsen *et al.*, 2020 ▸). Such long timescales are relevant for a number of different processes (Cipelletti *et al.*, 2000 ▸), among which the glass transition is a relevant example (Sidebottom *et al.*, 1993 ▸; van Megen *et al.*, 1998 ▸). The glass transition takes place, in fact, in a temperature range where the structural relaxation has a characteristic time of ∼100 s (Moynihan *et al.*, 1976 ▸; Böhmer *et al.*, 1993 ▸). This arbitrary definition finds its roots in the phenomenological observations that a system with that characteristic relaxation time is essentially frozen and its dynamical and mechanical responses resemble those of a solid.

In this work, the XPCS measurements were performed at beamline P10 of the PETRA III storage ring in Hamburg (Germany) (P10 beamline, 2023 ▸). A 8.05 keV X-ray beam (λ = 1.540 Å, horizontally polarized) was monochromated with a Si(111) channel-cut crystal and focused with beryllium compound refractive lenses onto a spot of 1.9 µm × 3.2 µm (V × H) full width at half-maximum (FWHM). The scattered intensity was collected using an EigerX4M detector mounted at 1.85 m from the sample on a goniometer arm moving in the horizontal plane and covering scattering angles up to 41°.

## 3. Flash calorimetry in a coherent X-ray scattering beamline

Calorimetric techniques are widely employed to study thermodynamic properties of materials. For example, during a phase transition such as crystallization, an amount of heat is released by the sample (the enthalpy of fusion), with the crystal featuring a lower heat capacity than the liquid. In a differential scanning calorimeter (DSC), this information is captured by measuring the heat flux required to increase the temperature of the sample by a given amount. Specifically, a typical DSC contains two twin cells with the same thermal properties; the sample is positioned in one of the cells and both cells are heated at a given rate, β. In power-compensated setups (Mathot *et al.*, 2011 ▸), the heating power delivered to the two cells is tuned such that the temperatures of the sample and reference cells are equal. The difference in power needed to achieve this is the measured power, Φ. Assuming an ideal calorimeter with perfectly symmetric cells, if no phase transition occurs in the temperature range of interest, the isobaric specific heat (*c*_P_) of the sample is related to the power by

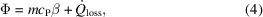

with *m* denoting the mass of the sample and 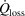 the power loss from the sample into the environment (see Appendix *B*1 for more details). From equation (4), one can see that a reduction in the mass of the sample is needed to achieve high heating/cooling rates with a reasonable power. Developments aimed in this direction resulted in fast scanning calorimeters in the early 2000s (Lai, 1998 ▸; Pijpers *et al.*, 2002 ▸) and eventually led to the advent of chip calorimeters (Lopeandía *et al.*, 2005 ▸; Mathot *et al.*, 2011 ▸). Chip calorimeters are built on the membrane of a micro-electronic mechanical system (MEMS) and samples of a few nanograms can be used; with such small masses, heating rates up to 10^6^ K min^−1^ have been achieved (Yi & LaVan, 2019 ▸).

In this work, we integrate a commercial Mettler Toledo Flash DSC 2+ calorimeter in a coherent scattering synchrotron radiation beamline enabling the investigation of thermal properties in combination with XPCS experiments. In its standard working condition, the Flash DSC chip (the calorimeter itself) is placed inside the chassis of the instrument, which contains all the electrical contacts and electronics to perform the DSC measurements as well as to communicate with the controller computer. The chassis is equipped with a temperature regulation system, a cryo-cooler (reaching a minimum temperature of 183 K) and a gas inlet to work in a controlled atmosphere with different gases (*e.g.* N_2_ or Ar). However, such a standard configuration of the calorimeter is not compatible with scattering experiments in a synchrotron beamline: the chassis of the instrument is neither transparent to X-rays nor compatible with vacuum conditions (crucial for low-background measurements).

Therefore, we have mirrored the electrical contacts in the chassis onto an external cell: the chip can then be coupled to the Flash DSC machine by means of a cable that connects the contacts on the chip to the chassis.

**Figure 1:**
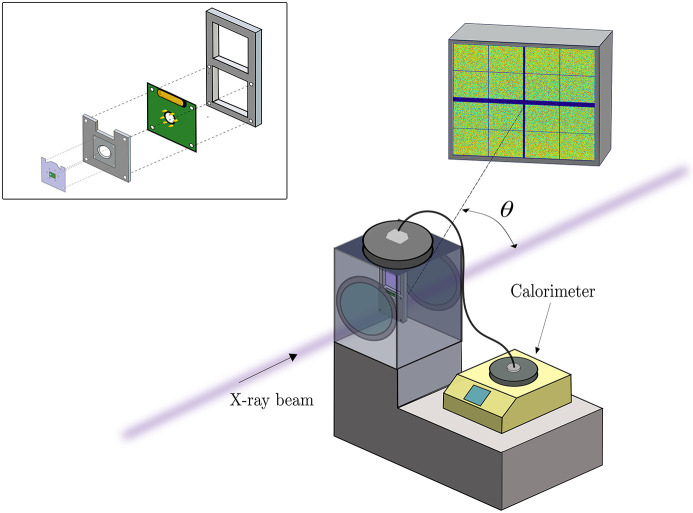
Scheme of the experimental setup developed for combined X-ray photon correlation spectroscopy and nano-calorimetry. The X-ray beam is focused onto a spot a few micrometres in size on the sample mounted on the calorimeter chip. The scattered radiation is collected with a 2D detector downstream of the experimental chamber. The chip’s electrical contacts are connected to the body of the calorimeter in order to control the temperature and perform thermal cycles (heating/cooling). Inset: zoom of the chip holder with illustration of the replicated contacts to connect the chip to the calorimeter.

In Fig. 1 ▸, we show a schematic diagram of the designed experimental setup. The chip is mounted on an external support compatible with a standard vacuum chamber at the P10 beamline at PETRA III, and a cable connects this holder to the calorimeter. The inset of Fig. 1 ▸ shows a sketch of the chip holder, where the contacts are reproduced on a printed circuit board (PCB) which, in turn, is fixed to the frame support, shaped to host the chip.

## 4. Background issues related to the chip membrane

In the Mettler Toledo chip calorimeter, the sample is placed on top of a thin membrane composed of a metallic thermally conductive layer (Al and Au for the standard UFS1 and high-temperature UFH1 chip, respectively) enclosed between two inert layers (silicon nitride and silicon oxide). The total thickness of this compound membrane is of the order of a few micrometres and the membrane is robust enough to allow manual positioning of the sample with the help of a microscope. Once on the active area, the sample can be prepared (*i.e.* attached by melting) and transported safely to the beamline.

**Figure 2:**
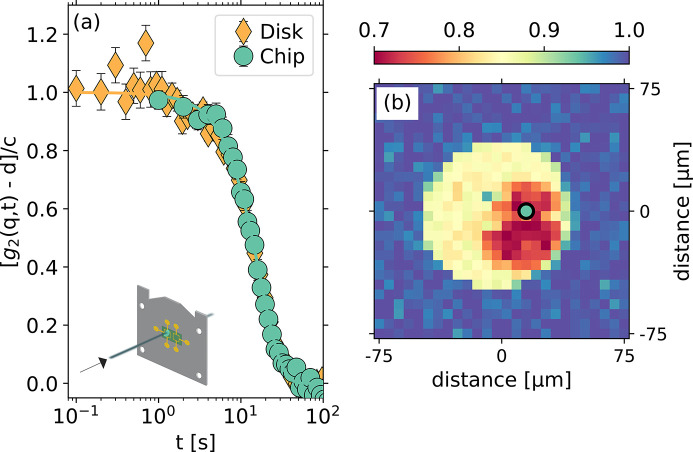
(*a*) Normalized intensity autocorrelation functions for two LiBO_2_ samples in transmission geometry: a disk, yellow diamonds, and a sample mounted on a UHF1 chip, green circles. Both measurements correspond to *q* = 17.0 ± 0.9 nm. The geometry used for the measurements on the sample on the chip is shown in the inset. (*b*) Transmission map (step size of 6 µm) of the LiBO_2_ sample corresponding to the measurements in (*a*) on a UFH1 chip. The yellow region corresponds to the active area of the chip, while the red region corresponds to the LiBO_2_ sample. The black-circled light-green circle shows the position where the measurement reported in (*a*) has been performed.

A possible drawback of the use of the chip calorimeter in combination with XPCS experiments is the background contribution introduced by the X-rays scattered by the chip membrane. We quantified this effect using a low atomic number, *Z*, glass: LiBO_2_. This is a worst-case scenario since the scattered intensity depends quadratically (at least at low *q*) on *Z*. We compared the signal scattered by the reference, *i.e.* the empty membrane, and by LiBO_2_ glass a few tens of micrometres thick, mounted on the sample membrane in transmission geometry [a sketch is shown in the inset of Fig. 2 ▸(*a*)]. The contribution of the membrane is about 10% of the total scattered intensity at the momentum transfer of *q* = 17 nm^−1^, *i.e.* at the first diffraction peak of LiBO_2_ (Dallari *et al.*, 2019 ▸; Martinelli *et al.*, 2023 ▸). Under these conditions, performing XPCS measurements on the empty membrane only gives rise to a noisy background with no measurable contrast in the correlation function.

To further demonstrate that the membrane does not contribute to spurious XPCS signal, we compare the results obtained for a stand-alone sample in a standard configuration with those obtained for a sample mounted on the calorimeter chip. In XPCS experiments, thin disk-shaped samples are routinely employed, with thicknesses of the order of 100 µm or less. The thickness is usually chosen as a compromise between getting a reasonable scattered signal and an acceptable contrast of at least a few percent at large scattering angles (Pintori *et al.*, 2019 ▸). In Fig. 2 ▸(*a*) we report the results obtained for a LiBO_2_ disk ∼150 µm thick (yellow diamonds) and for a LiBO_2_ sample mounted in transmission on the chip. The sample on the chip was prepared by cooling from 723 K at a rate of 0.15 K s^−1^ in order to mimic the protocol used to prepare the disk (see Appendix *A* [App appa]). Both measurements have been performed with a total exposure time of 120 s (frame-rate of 10 Hz and 1 Hz for the two measurements, respectively), and in Fig. 2 ▸(*a*) the intensity autocorrelation functions have been normalized to the contrast after baseline subtraction.

The auto-correlation functions reported in Fig. 2 ▸ have been collected at room temperature (297 K), and they show decorrelation at the time scale of 30 s. Note that the sample measured here is deep in its glassy state [the glass transition temperature is *T*_g_ ≃ 700 K (Martinelli *et al.*, 2023 ▸)] where the spontaneous dynamics are expected to be almost completely frozen. The effect we are probing is thus induced by the X-ray beam itself and is referred to as ‘beam-induced dynamics’ (Ruta *et al.*, 2017 ▸). It is observed in different oxide glasses such as borates and silicates (Ruta *et al.*, 2017 ▸; Pintori *et al.*, 2019 ▸; Dallari *et al.*, 2023 ▸; Martinelli *et al.*, 2023 ▸), and it is characterized by a relaxation time inversely proportional to the incident X-ray flux. This effect is due to electronic excitations that couple with the lattice and induce atomic displacements. Furthermore, it has been shown that this effect is in competition with the spontaneous structural relaxation close to the glass transition temperature (Pintori *et al.*, 2019 ▸), and that it depends on the total absorbed dose (Alfinelli *et al.*, 2023 ▸). Moreover, as long as the sample thickness is small compared with the attenuation length of the X-rays, the relaxation time does not depend on the sample thickness (Pintori *et al.*, 2019 ▸). This condition is well verified by both samples (in disk shape and on the calorimeter chip) used for our comparison.

Having clarified the origin of the signal in Fig. 2 ▸, we have verified that the relaxation times (τ_D_ = 27.0 ± 0.4 s and τ_O_ = 25.9 ± 0.7 s) and shape parameters (γ_D_ = 1.55 ± 0.05 and γ_O_ = 1.67 ± 0.10) for the disk-shaped sample and the sample mounted on the chip, respectively, are mutually consistent within one standard deviation. This is a clear indication of the good quality of the XPCS data collected using our calorimeter chip setup. Fig. 2 ▸(*b*) shows a transmission map of the sample membrane performed with a diode positioned downstream from the experimental chamber. The sample is clearly visible as the red region on the chip sensor. With this detailed map of the sample, the position of the X-ray beam on the sample can be easily chosen to avoid the thermocouples at the center of the chip.

**Figure 3:**
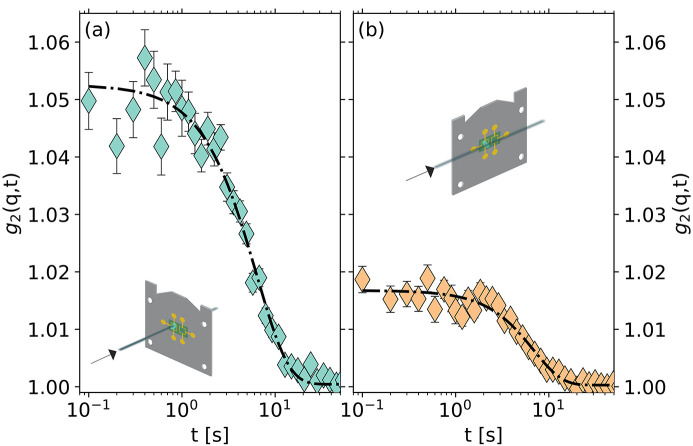
Intensity–intensity correlation function, *g*_2_(*q*, *t*), of a LiBO_2_ glass mounted on the UHF1 chip in ‘orthogonal’ (*a*) and ‘parallel’ (*b*) configuration. A difference in contrast is observable due to the different lengths of the samples along the beam, but the relaxation times (τ_O_ = 10.9 ± 0.3 s and τ_P_ = 11.2 ± 0.5 s for the orthogonal and parallel configurations, respectively) and the shape parameters (γ_O_ = 1.27 ± 0.08 and γ_P_ = 1.43 ± 0.13 for the orthogonal and parallel configuration, respectively) are compatible within one standard deviation. Both measurements have been performed on the first sharp diffraction peak at *q* = 17.0 ± 0.9 nm. The insets in the panels show the beam direction with respect to the calorimeter’s chip.

It is also interesting to explore different scattering configurations allowed by the chip calorimeter. In Fig. 3 ▸ we show the intensity–intensity correlation functions of a LiBO_2_ glass at room temperature in two different configurations (see insets). Both measurements reported in Fig. 3 ▸ have been carried out in transmission through the sample with the chip in different orientations with respect to the incident X-ray beam: in the first configuration, referred to as ‘orthogonal’, inset of Fig. 3 ▸(*a*), the X-ray beam goes through both sample and membrane; in the ‘parallel’ configuration, the X-ray beam is parallel to the chip plane but does not hit it, and only goes through the sample.

The samples have been prepared by quenching the supercooled liquid at *T* = 833 K down to room temperature with a cooling rate of 500 K s^−1^. In the orthogonal configuration (panel *a*), the X-ray beam goes through both sample and membrane, as shown in Fig. 2 ▸. In the parallel configuration, the sample is mounted on the back of the membrane, with the beam entering the sample parallel to the membrane without touching it, as shown in the inset of Fig. 3 ▸(*b*) (this would not be possible if we had mounted the sample on the standard side of the membrane due to the shape of the ceramic frame). The two measurements reported here have been performed keeping the same absorbed dose as it has been shown that the beam-induced dynamics depend on the total energy delivered to the sample by the X-ray beam (Martinelli *et al.*, 2023 ▸).

In Fig. 3 ▸, we show that the contrast for the two measurements is different, reflecting the difference in the scattering volumes. Indeed, for the sample mounted on the back of the chip sensor (the one in the parallel configuration), the length of the scattering volume along the X-ray beam is longer than for the sample utilized in the orthogonal configuration. However, the relevant dynamical parameters (the relaxation time and the shape parameter) are clearly in mutual agreement, indicating that both configurations can be equally well utilized and that the substrate does not play any critical role in the measurement. In the following, we will focus our attention on the orthogonal configuration, which is intrinsically simpler in terms of both sample preparation and XPCS measurements.

## 5. Calorimetric scans and use of the Flash calorimeter as a fast-responding furnace

We discuss now the use of the Flash DSC2+ chip calorimeter in vacuum conditions both to perform calorimetric scans and as a fast-responding furnace. One of the big advantages of this new generation of calorimeters is the possibility to study systems that easily crystallize, *e.g.* metallic glasses. We then decided to test our setup using a Pd-based metallic glass, Pd_42.5_Cu_30_Ni_7.5_P_20_, and we performed XPCS measurements in its supercooled liquid state.

**Figure 4:**
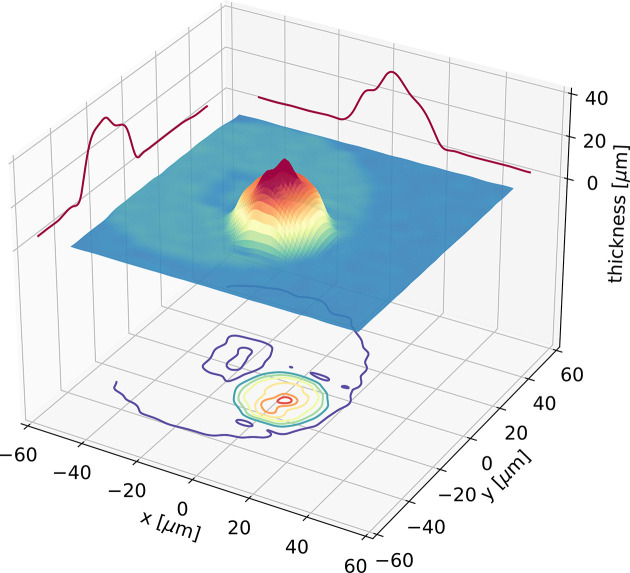
3D representation of the thickness of a Pd_42.5_Cu_30_Ni_7.5_P_20_ sample mounted on the chip calorimeter. The transmitted intensity is collected over a grid of equally spaced points (step size 4 µm). The thickness of the sample is then extracted knowing its density and composition. The 3D map has been smoothed and over-sampled (*i.e.* the experimental data have been interpolated with a spline) to increase its readability.

In Fig. 4 ▸, we report a map of a transmission measurement performed on a Pd_42.5_Cu_30_Ni_7.5_P_20_ glass mounted on a UFH1 chip. The measurement has been carried out scanning the sample in the plane orthogonal to the incident X-ray beam and the transmitted intensity (*I*) has been measured with a silicon diode. Since the incident flux, *I*_0_, is known, the thickness of the sample can be extracted from the Lambert–Beer equation,

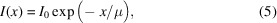

where *x* is the sample thickness and μ is the attenuation length. In the present case, μ = 7.78 µm for a density of ρ = 9.35 g cm^−3^ (Haruyama *et al.*, 2007 ▸). The thickness map displayed in Fig. 4 ▸ shows that the sample, after melting, has the shape of a small drop with a thickness of the order of ∼30 µm, a value that can be selected choosing the volume of the initial sample positioned on the chip.

**Figure 5:**
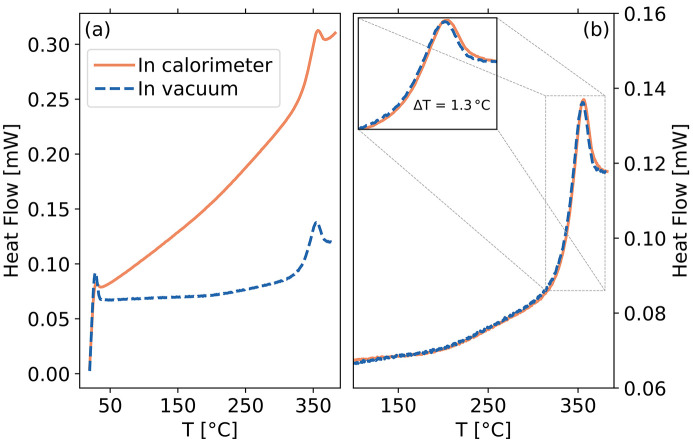
Heat flow measured for a sample of Pd_42.5_Cu_30_Ni_7.5_P_20_ with the Flash DSC 2+ calorimeter. (*a*) Raw data for a heating rate of β = 1000 K s^−1^ in standard conditions (full line), *i.e.* measured at ambient N_2_ pressure in the calorimeter chassis, and in vacuum inside the P10 experimental chamber (dashed line). (*b*) The thermograms of panel (*a*) are reported after data correction. Inset: zoom of the glass transition region, highlighting a small temperature difference (Δ*T* = 1.3 K) between the two thermograms.

In Fig. 5 ▸(*a*) we show some calorimetric scans performed under different conditions: we compare the calorimetry results placing the chip in the calorimeter chassis with those performed in vacuum. The continuous orange line is a heating scan (raw data) up to 660 K with β = 1000 K s^−1^ in the calorimeter chassis in N_2_ atmosphere. The chip has then been placed in our custom cell for XPCS measurements under vacuum (10^−6^ mbar), and the same scan has been repeated with the X-ray beam off (blue-dashed line). In both cases, the glass transition is clearly visible, despite the strong changes in the slope of the heat flow. The different slopes are due to different heat-loss mechanisms between the sample and the surrounding environment: under ambient pressure (in the chassis of the calorimeter), heat is dissipated via convection; in vacuum, heat-loss via convection is negligible, and conduction through the membrane is the dominating mechanism. As a consequence, the maximum cooling rate achievable in vacuum is smaller than the one that can be reached with an inert gas. If higher rates are required (not the case in this study), it is possible to fill the sample chamber with inert gas such as N_2_ for calorimetric scans.

In order to compare the scans collected in N_2_ atmosphere and in vacuum, we have to correct for the aforementioned heat-losses (Schick & Mathot, 2016 ▸; Abate *et al.*, 2022 ▸; Sonaglioni *et al.*, 2023 ▸). To this aim, we applied the ‘symmetric correction’ and an additional small baseline mismatch correction (Abate *et al.*, 2022 ▸) (more details in Appendix *B*1[App appb]). Fig. 5 ▸(*b*) shows the corrected data, where a very good agreement between the two curves can be seen, demonstrating the possibility to perform calorimetric scans in vacuum up to a rate of at least 1000 K s^−1^. Nano-calorimeters capable of working in vacuum have been developed some years ago (León-Gutierrez *et al.*, 2008 ▸) and are mainly used to probe samples at high heating rates. Our results show that the commercial chip-based calorimeter used here can also be operated in vacuum up to quite high rates.

In the inset of Fig. 5 ▸(*b*), a zoom of the heat flow curves close to *T*_g_ is reported. A small shift can be observed between the curves. This can be explained in terms of a thermal-lag effect: in chip-based calorimeters, the role of the gas as a heat-exchange medium is crucial (Schick & Mathot, 2016 ▸), and in the absence of the gas the thermal lag is expected to change and possibly require ‘non-symmetric’ correction terms (Sonaglioni *et al.*, 2023 ▸) which are partially taken into account here with the aforementioned baseline mismatch correction (Abate *et al.*, 2022 ▸).

**Figure 6:**
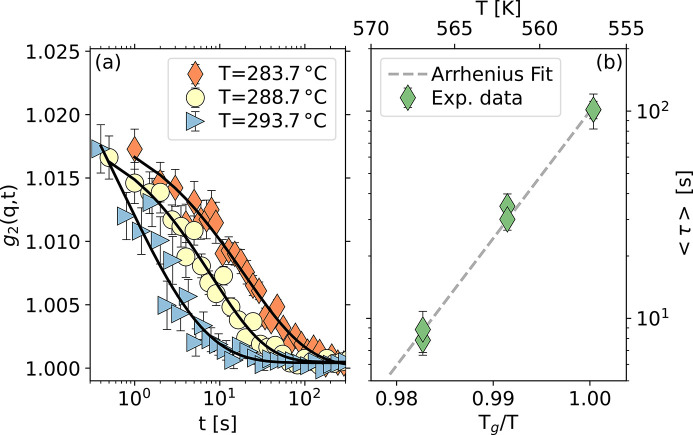
(*a*) Selection of intensity correlation functions measured at different temperatures (see legend) in the metallic glass Pd_42.5_Cu_30_Ni_7.5_P_20_ at *q* = (28.7 ± 0.4) nm^−1^. All the measured curves exhibit a stationary dynamics and a shape parameter γ < 1. (*b*) The average relaxation time is reported as a function of the inverse temperature scaled to the glass transition temperature, *T*_g_. In the temperature range investigated here, the data can be described by a simple exponential behavior (Arrhenius-like dependence, gray dashed line).

Lastly, we demonstrate the use of the chip calorimeter as a furnace in XPCS experiments using a Pd_42.5_Cu_30_Ni_7.5_P_20_ metallic glass sample mounted on an UFH1 chip in the orthogonal configuration geometry discussed above. We choose the following protocol to use the calorimeter as a furnace for a given temperature *T*: the sample is first heated at 1000 K s^−1^ up to 663 K (in the supercooled liquid state), then cooled down to *T* at 1000 K s^−1^ and then kept at *T* for a few minutes before starting the XPCS measurements. The measurements have been performed on the first diffraction peak of this sample, corresponding to *q* = 28.7 ± 0.4 nm^−1^. In Fig. 6 ▸(*a*) we show a selection of correlation functions measured at different temperatures. We performed two measurements at each temperature to check the reproducibility of the results. The curves have a stretched shape parameter (γ < 1), consistent with the fact that we are probing the supercooled liquid dynamics (Ruta *et al.*, 2012 ▸). We observe that the shape parameter does not show any clear trend with temperature and remains always stretched, with an average value γ = 0.63 ± 0.04.

In Fig. 6 ▸(*b*) we report the average relaxation time, 〈τ〉 = τ/γΓ(1/γ), with Γ the gamma function, as a function of the inverse temperature scaled to the glass transition temperature, *T*_g_. The temperature dependence of 〈τ〉 is well approximated by a simple exponential (Arrhenius) function in the small temperature range probed here (gray dashed line). Here, we define the glass transition temperature as the temperature where the average relaxation time 〈τ〉 = 100 s (Böhmer *et al.*, 1993 ▸). Taking into account the uncertainties deriving from the Arrhenius fit and the absolute temperature calibration of the chip, we estimate a glass transition temperature *T*_g_ = 557.3 ± 0.5 K, close to that previously measured on the same glass with standard calorimetry (Liu *et al.*, 2016 ▸), *T*_g_ = 560 K. This result demonstrates the robustness of the XPCS results but should be taken with care. In fact, in calorimetric measurements (*e.g.* DSC), the onset of the glass transition depends on the thermal protocol followed to prepare the glass (Evenson *et al.*, 2010 ▸; Gross *et al.*, 2017 ▸) and shifts to higher temperatures with increasing the cooling rate used to quench the melt. In addition, the relaxation time τ obtained in XPCS measurements depends on the exchanged momentum *q*, and is then in general different from the one estimated at *q* → 0 with the *T*_g_-shift method (Evenson *et al.*, 2010 ▸).

Finally, the average relaxation time data collected in the proximity of the glass transition temperature allow us to estimate the kinetic fragility 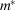 of our glass (Angell, 1995 ▸), 

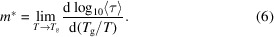

The value that we obtain is 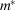 = 61 ± 2, slightly lower but in substantial agreement with one obtained from mechanical relaxation measurements (Liu *et al.*, 2016 ▸).

## 6. Conclusions

In conclusion, we have presented a simple setup that allows us to combine XPCS and calorimetric measurements using a nano-calorimeter. We have shown that mounting the sample on the chip of the calorimeter allows us to perform XPCS measurements in both orthogonal and parallel configuration. In the orthogonal configuration, the background coming from the chip membrane is negligible, and the results are comparable with those obtained using standard XPCS setups. Our setup also allows us to combine XPCS and calorimetric scans. In particular, we have shown that calorimetric measurements are possible with the chip in a vacuum environment suitable for XPCS experiments, at least up to the highest rates (1000 K s^−1^) investigated in this work. Finally, we have shown that the chip calorimeter can be used as a fast-responding furnace, allowing for the collection of XPCS data over an extended temperature range with quick temperature changes.

The key point to note here is that the sample size used in nano-calorimeters (∼10 µm) is perfectly matching the micrometre-sized beams nowadays routinely used in XPCS beamlines (P10 beamline, 2023 ▸) and the optimal sample thickness requirements given the partial coherence available at storage-rings beamlines (Madsen *et al.*, 2020 ▸).

## Appendix A Sample preparation

### A1. The lithium metaborate glass

The preparation of lithium metaborate (LiBO_2_) involved the use of lithium carbonate (Li_2_CO_3_, 99.99% purity) and anhydrous boron oxide (B_2_O_3_, 99% purity) as starting chemicals, both purchased from Sigma-Aldrich. As a first step, the powders were placed in an electric furnace (temperature stability ±1°C) and heated to 125°C for 24 h. This baking removes residual water and allows for a correct weight of the amount of Li_2_CO_3_ and B_2_O_3_. The powders were then mixed in the correct molar fraction, melted in an aluminium oxide (Al_2_O_3_) crucible and kept at 1000°C for 4.5 h. The melt was vitrified quenching it between stainless-steel plates preheated at 200°C. The obtained ingots were then annealed to release stresses for 6 h at 20°C below the tabulated glass transition temperature (Shelby, 1983 ▸). Since LiBO_2_ is hygroscopic, the obtained samples were kept in vacuum and prepared just before the XPCS experiments. For the XPCS measurements in the standard setup, the glass was cut and polished down to a thickness of ∼150 µm with silicon carbide sandpaper and ethanol as lubricant. The sample mounted on the Flash DSC calorimeter chip was instead powdered using a mortar until reaching grains of few tens of micrometres.

### A2. The Pd_42.5_Cu_30_Ni_7.5_P_20_ metallic glass

The ingot of Pd_42.5_Cu_30_Ni_7.5_P_20_ was re-melted several times under argon to ensure homogeneity. The metallic glass was then prepared in the shape of a ribbon by melt spinning under argon atmosphere. The copper wheel linear velocity was fixed to 40 m s^−1^, leading to ribbons of 30 ± 5 µm thickness and with an estimated quenching rate of 10^5^ to 10^6^ K s^−1^. The ribbons were cut into small pieces and one piece was then mounted on the UHF1 chip. The sample was melted on the chip cycling from room temperature up to 923 K with heating/cooling rates of 1000 K s^−1^. No onset of crystallization was observed in the collected calorimetric curves and no sign of Bragg peaks was detected during the XPCS measurements.

## Appendix B Flash DSC data treatment

### b1. Heat-flow baseline correction

Fast scanning nano-calorimeters can reach heating and cooling rates that are orders of magnitude higher than those of traditional differential scanning calorimeters. The data analysis required to convert the measured power to specific heat data has been discussed in specialized contributions (Schick & Mathot, 2016 ▸; Abate *et al.*, 2022 ▸; Sonaglioni *et al.*, 2023 ▸). Here we simply want to discuss the procedure used to obtain the data reported in Fig. 5 ▸(*b*).

The measured power is not simply proportional to the product of the cooling/heating rate and the thermal capacity of the sample under investigation, see equation (4) in the main text. In the range of temperatures usually investigated with nano-calorimeters, the power losses appearing in equation (4) are mainly related to the convective heat transfer between the sample and the surrounding gas.

The relevance of power losses in the measured power can be easily grasped from Fig. 5 ▸(*a*): the raw data obtained using the chip in the calorimeter chassis in N_2_ atmosphere show a roughly linear dependence of the power on the temperature. Clearly, since the isobaric specific heat is almost constant in the explored temperature range, the increase of the measured power is dominated by the contribution of  . Consistently, when the chip is in the experimental chamber in vacuum, Φ shows a mild dependence on the temperature below the glass transition because the convective heat transfer with the gas is strongly reduced.

We corrected for the power loss contribution using the so-called symmetric correction. The power balance in a heating and cooling cycle reads

where the subscript h (c) stands for heating (cooling). If one employs the same scanning rates in the two cycles, namely β|_h_ = −β|_c_, the first terms on the left hand side of the two equations above are opposite to one another. Moreover, the dissipated power depends only on the instantaneous temperature of gas and sample, hence  =  . Therefore,  can be calculated averaging the measured power in two symmetric cycles. The heat capacity of the sample can then be obtained.

Sometimes, non-reversible contributions to the measured power, *i.e.* terms that are not opposite to one another during symmetric heating and cooling cycles, have also to be considered, making the symmetric correction no longer fully satisfactory. This can be encountered, for example, when thermal lags are present (Sonaglioni *et al.*, 2023 ▸). In such situations, an additional ‘non-symmetric’ term must be included, which in first approximation corresponds to a small baseline rotation correction. A similar correction has been proposed by Abate *et al.* (2022 ▸), where the measured thermograms are appropriately rotated and shifted to correct for power losses.

In this spirit, the data reported in Fig. 5 ▸(*b*) have been first reduced using the symmetric correction, and a small residual baseline mismatch correction has then been applied. This last step has been implemented fitting the baseline of the calorimetric scans in vacuum and in N_2_ atmosphere to a line, as in the procedure of Abate *et al.* (2022 ▸), and using the fitted parameters to rotate the baseline of the scan collected in vacuum conditions to minimize the differences with the baseline of the scan collected in N_2_ atmosphere. These corrections have lead to the data reported in Fig. 5 ▸(*b*).

## References

[bb1] Abate, A., Cangialosi, D. & Napolitano, S. (2022). *Thermochim. Acta*, **707**, 179084.

[bb2] Alfinelli, E., Caporaletti, F., Dallari, F., Martinelli, A., Monaco, G., Ruta, B., Sprung, M., Zanatta, M. & Baldi, G. (2023). *Phys. Rev. B*, **107**, 054202.

[bb3] Angell, C. A. (1995). *Science*, **267**, 1924–1935. 10.1126/science.267.5206.1924 17770101

[bb4] Berne, B. J. & Pecora, R. (1976). *Dynamic Light Scattering: With Applications to Chemistry, Biology, and Physics.* Dover Publications.

[bb5] Böhmer, R., Ngai, K. L., Angell, C. A. & Plazek, D. J. (1993). *J. Chem. Phys.* **99**, 4201–4209.

[bb6] Cipelletti, L., Manley, S., Ball, R. C. & Weitz, D. A. (2000). *Phys. Rev. Lett.* **84**, 2275–2278. 10.1103/PhysRevLett.84.2275 11017262

[bb7] Dallari, F., Martinelli, A., Caporaletti, F., Sprung, M., Baldi, G. & Monaco, G. (2023). *Proc. Natl Acad. Sci. USA*, **120**, e2213182120. 10.1073/pnas.2213182120 PMC9926170 36608290

[bb8] Dallari, F., Pintori, G., Baldi, G., Martinelli, A., Ruta, B., Sprung, M. & Monaco, G. (2019). *arXiv*:1912.01943.

[bb9] Evenson, Z., Gallino, I. & Busch, R. (2010). *J. Appl. Phys.* **107**, 123529.

[bb10] Gross, O., Bochtler, B., Stolpe, M., Hechler, S., Hembree, W., Busch, R. & Gallino, I. (2017). *Acta Mater.* **132**, 118–127.

[bb11] Haruyama, O., Yokoyama, Y. & Inoue, A. (2007). *Mater. Trans.* **48**, 1708–1710.

[bb12] Lai, L. A. S. (1998). *Microscale Thermophys. Eng.* **2**, 11–19.

[bb13] León-Gutierrez, E., Garcia, G., Lopeandía, A. F., Fraxedas, J., Clavaguera-Mora, M. T. & Rodríguez-Viejo, J. (2008). *J. Chem. Phys.* **129**, 181101. 10.1063/1.3009766 19045378

[bb14] Liu, C., Pineda, E., Qiao, J. & Crespo, D. (2016). *J. Phys. Chem. B*, **120**, 2838–2844. 10.1021/acs.jpcb.5b11754 26916661

[bb15] Lopeandía, A. F., Cerdó, L. I., Clavaguera-Mora, M. T., Arana, L. R., Jensen, K. F., Muñoz, F. J. & Rodríguez-Viejo, J. (2005). *Rev. Sci. Instrum.* **76**, 065104.

[bb17] Madsen, A., Fluerasu, A. & Ruta, B. (2020). *Synchrotron Light Sources and Free-Electron Lasers: Accelerator Physics, Instrumentation and Science Applications*, 2nd ed., pp. 1989–2018. Springer.

[bb18] Martinelli, A., Caporaletti, F., Dallari, F., Sprung, M., Westermeier, F., Baldi, G. & Monaco, G. (2023). *Phys. Rev. X*, **13**, 041031.

[bb19] Mathot, V., Pyda, M., Pijpers, T., Vanden Poel, G., van de Kerkhof, E., van Herwaarden, S., van Herwaarden, F. & Leenaers, A. (2011). *Thermochim. Acta*, **522**, 36–45.

[bb21] Megen, W. van, Mortensen, T. C., Williams, S. R. & Müller, J. (1998). *Phys. Rev. E*, **58**, 6073–6085.

[bb22] Monaco, G., Fioretto, D., Masciovecchio, C., Ruocco, G. & Sette, F. (1999). *Phys. Rev. Lett.* **82**, 1776–1779.

[bb23] Monaco, G., Masciovecchio, C., Ruocco, G. & Sette, F. (1998). *Phys. Rev. Lett.* **80**, 2161–2164.

[bb24] Moynihan, C., Macedo, P., Montrose, C., Montrose, C., Gupta, P., DeBolt, M., Dill, J., Dom, B., Drake, P., Easteal, A., Elterman, P. B., Moeller, R. P., Sasabe, H. & Wilder, J. A. (1976). *Ann. N. Y. Acad. Sci.* **279**, 15–35.

[bb20] P10 beamline (2023). *P10 Coherence Applications Beamline*, https://photon-science.desy.de/facilities/petra_iii/beamlines/p10_coherence_applications.

[bb25] Perakis, F. & Gutt, C. (2020). *Phys. Chem. Chem. Phys.* **22**, 19443–19453. 10.1039/d0cp03551c 32870200

[bb26] Pijpers, T. F., Mathot, V. B., Goderis, B., Scherrenberg, R. L. & van der Vegte, E. W. (2002). *Macromolecules*, **35**, 3601–3613.

[bb27] Pintori, G., Baldi, G., Ruta, B. & Monaco, G. (2019). *Phys. Rev. B*, **99**, 224206.

[bb28] Rosenthal, M., Doblas, D., Hernandez, J. J., Odarchenko, Y. I., Burghammer, M., Di Cola, E., Spitzer, D., Antipov, A. E., Aldoshin, L. S. & Ivanov, D. A. (2014). *J. Synchrotron Rad.* **21**, 223–228. 10.1107/S1600577513024892 24365940

[bb29] Ruta, B., Chushkin, Y., Monaco, G., Cipelletti, L., Pineda, E., Bruna, P., Giordano, V. & Gonzalez-Silveira, M. (2012). *Phys. Rev. Lett.* **109**, 165701. 10.1103/PhysRevLett.109.165701 23215091

[bb30] Ruta, B., Zontone, F., Chushkin, Y., Baldi, G., Pintori, G., Monaco, G., Rufflé, B. & Kob, W. (2017). *Sci. Rep.* **7**, 3962. 10.1038/s41598-017-04271-x PMC5479813 28638053

[bb31] Sandy, A. R., Zhang, Q. & Lurio, L. B. (2018). *Annu. Rev. Mater. Res.* **48**, 167–190.

[bb32] Schawe, J. E. & Pogatscher, S. (2016). *Fast Scanning Calorimetry*, pp. 3–80. Springer.

[bb33] Schick, C. & Mathot, V. (2016). *Fast Scanning Calorimetry.* Springer.

[bb34] Shelby, J. (1983). *J. Am. Ceram. Soc.* **66**, 225–227.

[bb35] Sidebottom, D., Bergman, R., Börjesson, L. & Torell, L. (1993). *Phys. Rev. Lett.* **71**, 2260–2263. 10.1103/PhysRevLett.71.2260 10054628

[bb36] Sonaglioni, D., Tombari, E. & Capaccioli, S. (2023). *Thermochim. Acta*, **719**, 179385.

[bb37] Williams, G. & Watts, D. C. (1970). *Trans. Faraday Soc.* **66**, 80–85.

[bb38] Willmott, P. (2019). *An Introduction to Synchrotron Radiation: Techniques and Applications.* John Wiley & Sons.

[bb39] Xiao, K., Gregoire, J. M., McCluskey, P. J., Dale, D. & Vlassak, J. J. (2013). *J. Appl. Phys.* **113**, 243501. 10.1063/1.4806972 PMC3676369 23825802

[bb40] Yi, F. & LaVan, D. A. (2019). *Appl. Phys. Rev.* **6**, 031302.

[bb41] Zheng, Q., Zhang, Y., Montazerian, M., Gulbiten, O., Mauro, J. C., Zanotto, E. D. & Yue, Y. (2019). *Chem. Rev.* **119**, 7848–7939. 10.1021/acs.chemrev.8b00510 31120738

